# 2342 Protein production as an early pharmacodynamics biomarker for RNA-targeting therapies

**DOI:** 10.1017/cts.2018.110

**Published:** 2018-11-21

**Authors:** Wade K. Self, Kathleen Schoch, James Bollinger, Tracy Cole, Holly Kordasiewicz, Randall Bateman, Timothy Miller

**Affiliations:** Institute of Clinical and Translational Sciences, Washington University in St. Louis

## Abstract

OBJECTIVES/SPECIFIC AIMS: We aimed to develop an assay to measure new protein synthesis after Antisense Oligonucleotide treatment, which we hypothesized to be the earliest biochemical identification of RNA-targeting therapy efficacy. METHODS/STUDY POPULATION: We treated 2 transgenic animal models expressing proteins implicated in neurodegenerative disease: human tau protein (hTau) and human superoxide dismutase 1 (hSOD1), with ASO against these mRNA transcripts. Animals received isotope-labeled 13C6-Leucine via drinking water to label newly synthesized proteins. We assayed target protein synthesis and concentration after ASO treatment to determine the earliest identification of ASO target engagement. RESULTS/ANTICIPATED RESULTS: hTau ASO treatment in transgenic mice lowered hTau protein concentration 23 days post-treatment in cortex (95% CI: 0.05%–64.0% reduction). In the same tissue, we observed lowering of hTau protein synthesis as early as 13 days (95% CI: 29.4%–123%). In hSOD1 transgenic rats, we observed lowering of 13C6-leucine-labeled hSOD1 in the cerebrospinal fluid 30 days after ASO treatment compared with inactive ASO control (95% CI: 12.0%–48.4%). DISCUSSION/SIGNIFICANCE OF IMPACT: In progressive neurodegenerative diseases, it is crucial to develop measurements that identify treatment efficacy early to improve patient outcomes. These data support the use of stable isotope labeling of amino acids to measure new protein synthesis as an early pharmacodynamics measurement for therapies that target RNA and inhibit the translation of proteins.

